# Electrospun Silk Fibroin-CNT Composite Fibers: Characterization and Application in Mediating Fibroblast Stimulation

**DOI:** 10.3390/polym15010091

**Published:** 2022-12-26

**Authors:** Rathnayake A. C. Rathnayake, Shinhae Yoon, Shuyao Zheng, Elwin D. Clutter, Rong R. Wang

**Affiliations:** Department of Chemistry, Illinois Institute of Technology, Chicago, IL 60616, USA

**Keywords:** electrospinning, biopolymer, silk fibroin, CNT, fibroblast stimulation

## Abstract

Electrospinning is a simple, low-cost, and highly efficient technique to generate desirable nano/microfibers from polymer solutions. Silk fibroin (SF), a biopolymer found in Bombyx mori cocoons, has attracted attention for various biomedical applications. In this study, functionalized CNT was incorporated in SF to generate biocomposite fibers by electrospinning. The electrospun (E-spun) fibers were well aligned with morphology mimicking the locally oriented ECM proteins in connective tissues. While as-spun fibers dissolved in water in just two minutes, ethanol vapor post-treatment promoted β-sheet formation leading to improved fiber stability in an aqueous environment (>14 days). The addition of a minute amount of CNT effectively improved the E-spun fiber alignment and mechanical strength while retained high biocompatibility and biodegradability. The fibers’ electrical conductivity increased by 13.7 folds and 21.8 folds, respectively, in the presence of 0.1 w% and 0.2 w% CNT in SF fibers. With aligned SF-CNT 0.1 % fibers as a cell culture matrix, we found electrical stimulation effectively activated fibroblasts from patients of pelvic organ prolapse (POP), a connective tissue disorder. The stimulation boosted the fibroblasts’ productivity of collagen III (COLIII) and collagen I (COLI) by 74 folds and 58 folds, respectively, and reduced the COLI to COLIII ratio favorable for tissue repair. The developed material and method offer a simple, direct, and effective way to remedy the dysfunctional fibroblasts of patients for personalized cell therapeutic treatment of diseases and health conditions associated with collagen disorder.

## 1. Introduction

Biopolymer scaffolds with tailored biochemical composition and biophysical characteristics are in high demand for tissue regeneration. Among a variety of techniques to generate biomaterial scaffolds, electrospinning has gained considerable interest. The process is remarkably efficient, rapid, inexpensive, and the electrospun (E-spun) fibrous architecture mimics the native protein fibrils in an extracellular matrix to support cell growth and tissue remodeling. Electrospinning is also convenient to produce composite fibers by incorporating other components. Biocomposite matrices capable of coaxing cell development are highly desirable for reconstruction of lesioned tissues in tissue engineering and cell therapeutics.

Silk has become a favorable material in biomedical research due to its biocompatibility, biodegradability, oxygen and nutrient permeability, low immunogenicity, and robust mechanical performance [1]. Generating silk composites to enhance the functionality is a subject of particular interest to our research group. In our previous work, aligned collagen-silk composite fibers with controllable diameters and uniform morphology were produced by electrospinning [2,3]. Parameters and spinning conditions were optimized to achieve fibers with balanced biochemical and biophysical properties to induce rapid neural differentiation of stem cells. We also modulated matrix properties by incorporating carbon nanotube (CNT) into collagen to generate collagen-CNT composite fibers [4,5]. It was found that incorporating a low dose of oxidized CNT into collagen greatly accelerated stem cell differentiation due to CNT’s impact on collagen fiber structure and stiffness [4,5]. We also explored the addition of CNT to spider silk protein and capitalized on the increased electrical conductivity of the biocomposite fibers to aid in fibroblast activation by electrical stimulation [6,7].

Since natural spider silk is difficult to harvest in large amount due to spiders’ low silk output and limitation of farming, spider silk proteins isolated from milk of transgenic goats were used in our studies [2,6,7]. However, the process of acquiring the proteins is at high cost, labor-intensive and time-consuming. Silk fibroin (SF) isolated from the cocoons of Bombyx mori silkworm offers a great alternative and has attracted increasing interest due to its compatible quality as well as its abundance and processing ease owing to the mature sericulture industry [1,8]. These properties have paved way for variety of its applications as a unique material in sensor development [9,10], an agent for drug delivery [8,11] and a scaffold for tissue engineering [12,13]. In this study, we generated aligned, free-standing SF-CNT composite fibers by electrospinning and explored the application in stimulating fibroblasts extracted from tissues of pelvic organ prolapse (POP) patients.

POP is characterized by weakening of the pelvic floor connective tissues and loss of support for pelvic organs. Collagen is the predominant structural protein in the tissues. Consistent with reports in the literature [14,15,16], reduced collagen content, increased collagen I (COLI) to collagen III (COLIII) ratio and abnormal nanoscopic to microscopic structures of fibrillar collagen in pelvic floor connective tissues of POP patients were observed in our previous studies [17,18,19]. These are closely relevant to the cellular deficit of patients’ fibroblasts, which synthesize collagen and relevant metabolites to regulate the structure and function of the fibrous matrix in connective tissues [10,14,16]. The patients’ cells displayed poor contractility and low collagen productivity. Here, we employed the E-spun SF-CNT fibers with desirable functionalities to mediate electrical stimulation of patient’s cells to restore and improve the cell function, serving for personalized cell therapeutic treatment.

## 2. Materials and Methods

### 2.1. Silk Fibroin Regeneration

Silk fibroin was extracted and purified from *Bombyx mori* silkworm cocoons (Aurora Silk, Portland, OR, USA) following the protocol reported by Wray et al. [20]. In brief, cocoons were cut into small pieces and boiled for 1 h in 0.02 M Na_2_CO_3_ aqueous solution to remove sericin, a process called degumming. The degummed fibers were rinsed thoroughly with deionized water, air dried, then added to 9.3 M LiBr at 60 °C for 3 h to dissociate the silk fibers and increase the water solubility. The SF solution was dialyzed against deionized water in a cellulose tubular membrane (MWCO 6–8 kDa, Fisher Scientific, Pittsburgh, PA, USA) for 3 days to eliminate residual ions from the solution. After dialysis, the SF solution was centrifuged at 9700 rpm and 4 °C for 20 min to remove insoluble particulates. The supernatant was frozen at −80 °C overnight before it was lyophilized using a VirTis Freezemobile lyophilizer (SP Industries, Gardiner, NY, USA) to produce a white, soft, fibrous dry powder. SF was then dissolved in 1,1,1,3,3,3-hexafluoro-2-propanol (HFIP) (Oakwood Chemical, West Columbia, SC, USA) for electrospinning.

### 2.2. Preparation of E-Spun SF-CNT Fibers

To generate SF-CNT scaffolds at various CNT percentage, oxidized single-walled CNTs (cat# US4824, US Research Nanomaterials Inc., Houston, TX, USA) were dispersed in HFIP to form a 2 mg/mL suspension. A minute amount of the suspension was added to 100 mg/mL SF in HFIP to form mixtures containing 0.02 wt% to 0.2 wt% CNT in SF, designated as SF-CNT 0.02% to SF-CNT 0.2%. Electrospinning was carried out at a flow rate of 0.8 mL/h using a home-built electrospinning system [2,3,7]. A high electric potential of 25 kV was applied to the protein droplet at the tip of an 18 Gauge blunt needle (Global Medical Products, Port St Lucie, FL, USA) to produce aligned, high-density fibers across two parallel metal plates, placed 10 mm apart. The free-standing fibers were then transferred to a pre-cut glass slide, then dried in a vacuum chamber overnight at room temperature to remove residual HFIP.

To study the effect of protein solution viscosity on fiber alignment and diameter, viscosity of SF solutions in the absence and presence of CNT was measured using a DV3T Rheometer (AMETEK Brookfield, Middleborough, MA, USA) at 25 °C. For each sample, viscosity was measured using a 0.5 mL solution at spindle speeds of 5–13 rpm, and the mean value was derived from three replicates.

As-spun fibers can quickly dissolve in water, therefore, cannot serve as a matrix to support cell growth. To improve the aqueous stability of the E-spun fibers, ethanol post-treatment was carried out by exposing the as-spun fibers to the vapor of 90% *v/v* ethanol for 12 h. For cell culture, the fibers were sterilized by immersing in 70% *v/v* ethanol at room temperature for 10 min, followed by 3 rinses with deionized water.

### 2.3. Aqueous Stability of Fibers and Biodegradation Assays

To test aqueous stability of as-spun and post-treated fibers, pre-weighed 3–5 mg SF-CNT fibers containing 0.0–0.5 wt% CNT in SF were immersed in deionized water at room temperature for 2 min. After supernatant was removed, the samples were vacuum dried overnight before they were weighed again to determine the remaining dry weight. The stability was evaluated by the percentage of weight recovery:

(1) $$Weight\;Recovery\;\%=\left(\frac{Remaining\;dry\;weight\;of\;fibers}{Initial\;dry\;weight\;of\;fibers}\right)\times 100\%$$

The post-treated fibers’ biodegradability was also examined. Pre-weighed (3–5 mg) fibers were placed in 100 U/mL Collagenase Type I (Gibco, Big Cabin, OK, USA) in HBSS solution and incubated in a shaker at 37 °C and 120 rpm. Data were collected on Days 1, 2, 3, 7, 10, and 14. At each time point, a sample was centrifuged at 12,000 rpm for 3 min. After removal of supernatant, the precipitate was washed with DI water 3 times, then vacuum dried overnight before it was weighed again to determine the remaining dry weight. The degree of fiber degradation was evaluated by the percentage of weight loss:

(2) $$Weight\;Loss\;\%=\left(\frac{Initial\;weight\;of\;fibers-Final\;weight\;of\;fibers}{Initial\;weight\;of\;fibers}\right)\times 100\%.\;$$

### 2.4. Characterization of Fibers’ Physical Properties

Fiber conductivity was derived from voltage-current curves of low-density fibers aligned between two pure gold electrodes using a patch clamp amplifier (Axopatch 200B Molecular Devices, Sunnyvale, CA, USA), as reported previously [6,7]. The number of fibers was determined in optical images acquired by a Leica DMi8 microscope (Leica Microsystems, Wetzler, Germany).

Fiber alignment was analyzed based on phase images of SF-CNT fibers. A 2-D fast Fourier transform (2-D FFT) approach [21] was applied to process 2048 × 2048 pixels of 8-bit gray-scale images using ImageJ software (NIH free download) supported by an oval profile plugin. Alignment index was defined by the ratio of the highest and the lowest peak values in an FFT plot [2,6].

Fiber diameter was determined by atomic force microscopy (AFM) using a multimode Nanoscope IIIa AFM (Veeco Metrology, Santa Barbara, CA, USA) equipped with a J-scanner. Images of E-spun fibers were collected in air tapping mode using silicon probes (K-Tek Nanotechnology, Wilsonville, OR, USA) at a resonance frequency of ~170 kHz, and analyzed using the Nanoscope analysis software to evaluate fiber diameter. Young’s modulus of SF and SF-CNT fibers was derived from force-distance curves collected using Si_3_N_4_ probes in fluid contact mode in PBS buffer [17,18]. From each force–distance curve, Young’s modulus was evaluated using a Hertzian model with the AFM tip modeled as a nano-indenter to probe a one-dimensional material [17,22]. The mean value was calculated based on a minimum of 50 force curves collected on various fibers at 2–4 randomly selected regions of fiber matrices.

### 2.5. Spectroscopic Analysis

Surface-enhanced Raman spectroscopic (SERS) analysis of E-spun fibers on a gold-coated glass substrate was carried out using a Renishaw Invia Raman Microscope (West Dundee, IL, USA) with a 90 mW laser at an excitation wavelength of 785 nm. Each spectrum was recorded via a 50× objective lens at 80 s exposure and 5-time accumulation. The spectra were normalized with respect to the 1450 cm^−1^ peak, which was assigned for asymmetric methyl deformation and its intensity is independent of protein secondary structures [23].

Attenuated total reflection Fourier transform infrared (ATR- FTIR) spectra of E-spun fibers were collected using a Thermo Nicolet Nexus 470 FTIR spectrometer (Madison, WI, USA), equipped with a single-bounce diamond ATR crystal accessory. The spectra were collected at a nominal resolution of 4 cm^−1^. Peak deconvolution of the amide I region was carried out following the procedure developed by Kreplak et al. [24] to evaluate the protein fibers’ secondary structural changes (Figure S1).

### 2.6. Cell Extraction, Culture, and Testing

Vaginal fibroblasts of a Stage III POP patient with severe posterior prolapse were used in this study. The biopsy was harvested at the posterior fornix of the patient following approved IRB protocol (#19071703-IRB01). Fibroblast extraction was carried out according to the previously established protocol [6,25]. In brief, fresh tissue fragments were placed in a 4 mL serum-free cell culture medium with 0.003 g/mL collagenase (Worthington Biochemicals, Lakewood, NJ, USA) and 0.048 g/mL of hyaluronidase (Worthington Biochemicals, Lakewood, NJ, USA), and incubated in a shaker for 30 min at 37 °C. The supernatant was then removed by aspiration. These steps were repeated twice. The remaining tissue fragments were further digested overnight, and the cells in the supernatant were collected by precipitating the cells with a centrifuge at 15 k rpm for one minute. The pellet was suspended in DMEM supplemented with 10% (*v*/*v*) fetal bovine serum (Corning, Manassas, VA, USA), 1% (*v*/*v*) MEM non-essential amino acid (Gibco, Carlsbad, CA, USA), 1% (*v*/*v*) MEM vitamin (Gibco, Carlsbad, CA, USA) and 2% (*v*/*v*) penicillin-streptomycin (10,000 U/mL) (Gibco, Carlsbad, CA, USA), plated onto a culture dish, and incubated for 24 h in 5% CO_2_ at 37 °C. The unattached cells were then removed, and the attached fibroblasts kept growing in a complete medium in 5% CO_2_ at 37 °C. Fresh medium was replaced every two days. At confluence, cells were detached using trypsin EDTA (0.25%) (Gibco, Carlsbad, CA, USA) and seeded on target matrices at a seeding density of 8000 cells/cm^2^. Cells in passage 3 were used in this study.

Cell viability and proliferation tests were carried out by CellTiter 96^®^ Aqueous One Solution Cell Proliferation Assay (MTS) (Promega, Madison, WI, USA) following the manufacturer’s instruction. Fibroblasts seeded on SF-CNT fibers, plastic, or gelatin coated Petri dishes were cultured and examined at different time points. Percentage of metabolically active cells on various matrices was derived with respect to the culture on Petri dish. Cell viability was evaluated 8 h post-plating based on the percentage of live cells with respective to the culture on Petri dish [2,6].

### 2.7. Electrical Stimulation of Cells

In vitro cell stimulation was carried out using a self-designed, homemade electrical stimulation device [7] with modification to generate a unform and stable electric field. Specifically, the device was powered by a 9 V battery and generate a 60 Hz square-wave (0 V to 0.45 V) between two gold-coated glass electrodes (2.0 cm × 1.0 cm), which were 0.5 cm apart in cell culture medium. After cells were seeded on the aligned fibers and cultured for 12 h, they were placed in the electric field for 10 min stimulation, followed by 10 min rest (to avoid protein aggregation) and another 10 min stimulation. The electric field was applied in the direction of fiber alignment. The whole process was carried out in an incubator at 5% CO_2_ and 37 °C. The cells were further cultured for 48 h before testing the stimulation effect.

### 2.8. Optical Imaging

Expression of α-smooth muscle actin (α-SMA) in unstimulated and stimulated cells was examined by immunofluorescence imaging using a Leica DMi8 microscope. Cells grown on various matrices were fixed by methanol for 10 min at –10 °C followed by air-dry, then blocked with 1% bovine serum albumin (Sigma-Aldrich, St. Louis, MO, USA) in PBS for 30 min. The cells were then incubated with the primary antibody (#PIMA511547, Thermofisher, Waltham, MA, 1:200 dilution) overnight at 4 °C. The secondary antibody, conjugated with Alexa Fluor 488 fluorescent dye (#A21206, Invitrogen, Carlsbad, CA, USA, 1:200 dilution), was incubated with the samples for 1 h in a dark, humidified chamber. The nuclei were stained with DAPI at a 1:1000 dilution for 10 min at room temperature. Both phase and fluorescent images were collected. Imaging parameters, such as the exposure time, gain values, image size, and the magnification, were kept constant throughout the entire study for comparative study of all samples. Quantitative analyses of fluorescence intensity and mean nuclear area were carried out using ImageJ software.

Change of cell morphology was examined before and after electrical stimulation. Cell length and cell area were measured from 10× phase-contrast images using ImageJ software. The effective cell width was determined using the ratio of cell area to cell length. Cell polarity was characterized by the cell length-to-width ratio:

(3) $$Cell\;Polarity=\frac{Cell\;length}{Effective\;cell\;width}=\frac{{\left(Cell\;length\right)}^{2}}{Cell\;area}$$

### 2.9. Total RNA Extraction and RT-qPCR Analysis

Total RNA was extracted from fibroblasts cultured on various matrices using a PureLink^®^ RNA Mini Kit (Ambion, Grand Island, NY, USA). The yield of the extracted total RNA was determined by Nanodrop (Thermo Scientific NanoDrop 1000, Waltham, MA, USA). Reverse-transcription was carried out using an APExBIO First-Strand cDNA Synthesis kit (APExBIO Technology, Houston, TX, USA). RT-qPCR was performed using a Bio-Rad MyiQ single-color real-time PCR machine with PrimeTime Gene Expression master mix and primers (Integrated DNA Technologies, Coralville, IA, USA). Primers against the following genes were used in this study: COL1A1 (Hs.PT.58.15517795), COL3A1 (Hs.PT.58.4249241)), MMP2 L1 (Hs.PT.58.38701397) and ACTA2 (Hs405785835). GAPDH (Hs99999905_m1) was used as an endogenous reference. Data analysis was carried out using the 2^−ΔΔCT^ method for relative quantification based on three replicates.

## 3. Results

### 3.1. Characterization of As-Spun Silk-CNT Fibers

**Figure 1:**
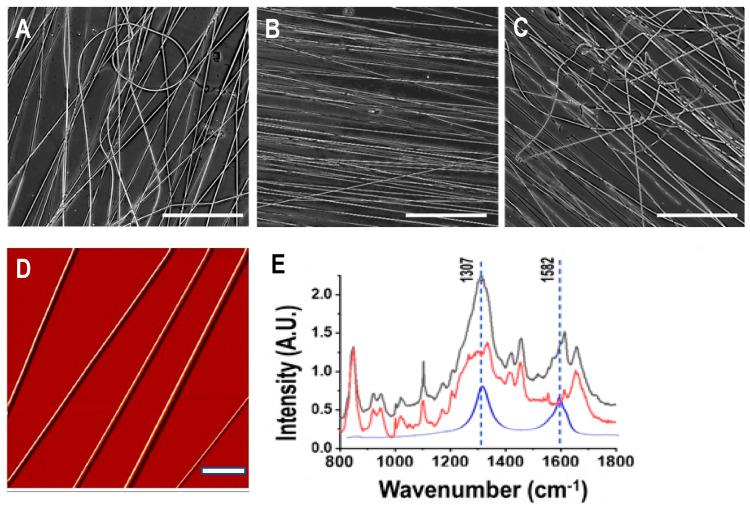
Structures of as-spun fibers. (**A**–**C**) Optical images of fibers of pure SF (**A**), SF-CNT 0.1% (**B**) and SF-CNT 0.25%. Bar size: 250 µm. (**D**) AFM image of SF fibers, demonstrating uniformity of each fiber along the fiber axis. Bar size: 10 µm. (**E**) Raman spectra of SF fibers (red) SF-CNT 0.1% fibers (black) and pure CNT (blue).

Figure 1A–C show optical images of as-spun pure SF, SF-CNT 0.1% and SF-CNT (0.25%) fibers collected on glass substrates. While fibers of all types are primarily aligned, SF-CNT 0.1% fibers are best aligned. Mis-aligned, curved fibers were frequently observed in pure SF and SF-CNT (0.25%) fiber matrices. As shown in the high-resolution AFM image (Figure 1D), SF fibers are smooth and uniform along the fiber axis. Distortion and defects were observed in SF-CNT fibers at higher CNT percentage.

Raman spectra of SF fibers, SF-CNT 0.1% fibers and CNT are shown in Figure 1E. The CNT spectrum displayed the characteristic Raman shifts at 1307 cm^−1^ (D-band) and 1582 cm^−1^ (G-band). While the spectrum of SF fibers (red) is complex in the spectral range, prominent peaks at 1307 cm^−1^ and 1582 cm^−1^ are apparent in the spectrum of SF-CNT fibers (black), attesting the integration of CNT in the SF protein fibers. In addition to the characteristic peaks associated with C-C stretches (900–1100 cm^−1^), amide III (1200–1300 cm^−1^), and amide I (1600–1700 cm^−1^), a strong peak at 850 cm^−1^, correlated with tyrosine residues in silk protein [26], was also observed.

### 3.2. Effect of Ethanol Post-Treatment

**Figure 2:**
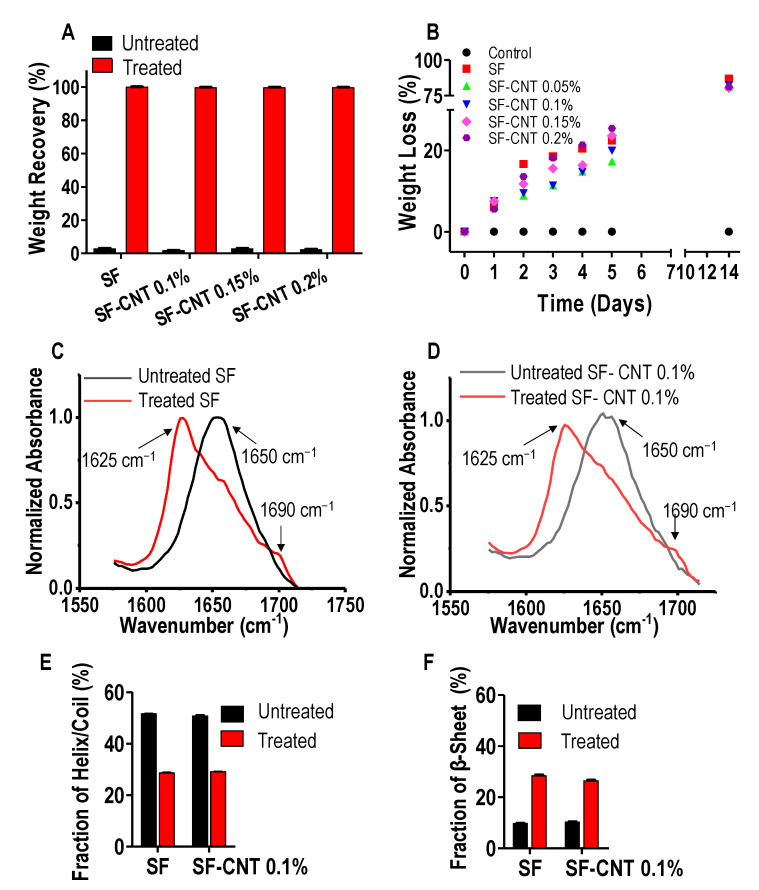
Post-treatment effect on E-spun fibers aqueous stability, degradability and secondary structural transition. (**A**) Aqueous stability of untreated (black) and treated (red) SF and SF-CNT fibers characterized by weight recovery after fiber immersion in DI water for 2 min at room temperature. (**B**) Weight loss of treated SF and SF-CNT fibers against collagenase digestion time. Weight loss of treated SF fibers in the absence of enzyme was a control. (**C**,**D**) ATR-FTIR spectra of untreated (black) and treated (red) SF (**C**) and SF-CNT 0.1% (**D**) fibers in amide I region. (**E**,**F**) Change of fractional distribution of α-helix/random coil (**E**) and β-sheet (**F**) in untreated (black) and treated (red) fibers. The difference is statistically significant (*p* < 0.001) for untreated and treated fibers.

As-spun SF and SF-CNT fibers were found unstable in aqueous solution. As shown in Figure 2A, after the fibers were immersed in DI water at room temperature, the fibers dissolved completely in 2 min. With ethanol post-treatment, the fibers showed negligible weight loss. Even after 14 days in cell culture medium, the fibers remained intact (see control in Figure 2B). Treated fibers are stable, durable, and capable of supporting long-term cell culture. As a tissue-engineering scaffold, degradability is also essential and was examined using an in vitro collagenase degradation assay. As shown in Figure 2B, the weight loss of post-treated SF and SF-CNT fibers gradually increased with digestion time regardless of CNT percentage in the fibers, and only 23% fibers remained after 14 days. In contrast, a negligible change of weight was observed when post-treated SF fibers were immersed in the same medium in the absence of collagenase (control). The result suggests that, while the post-treatment improved water stability of the fibers, the treated fibers are enzymatically degradable, rendering the fibers good candidates of implant constructs.

Ethanol treatment is known to induce secondary structural change of silk proteins [27]. FTIR spectrum at the amide I region (1585–1710 cm^−1^) is mainly associated with the carbonyl group of the polypeptide backbone and is a sensitive marker for proteins’ secondary structure [25,26]. The peak centered at 1650 cm^−1^ is characteristic of α-helices and random coils, and the peaks at 1620 cm^−1^ and 1690 cm^−1^ are characteristic of β-sheets [3,28]. As shown in Figure 2C,D, ethanol treatment induced a marked decrease of the 1650 cm^−1^ peak and a significant increase of the 1620 cm^−1^ and 1690 cm^−1^ peaks, suggesting the helix/coil to β-sheet transition in both SF and SF-CNT fibers [29]. The changes were quantitatively analyzed by spectral deconvolution (Figure S1), and summarized in Figure 2E,F. Apparently, the helix/coil structure was predominant in as-spun SF and SF-CNT fibers; the β-sheet component was almost tripled by ethanol treatment. Therefore, ethanol treatment effectively converted the helix/coil structure to the β-sheets, which contributes to the mechanical strength and water stability of the post-treated fibers. The secondary structural change was further evidenced by Raman spectral analysis in amide I and amide III regions (Figure S2).

### 3.3. Effect of CNT on Fiber Properties

**Figure 3:**
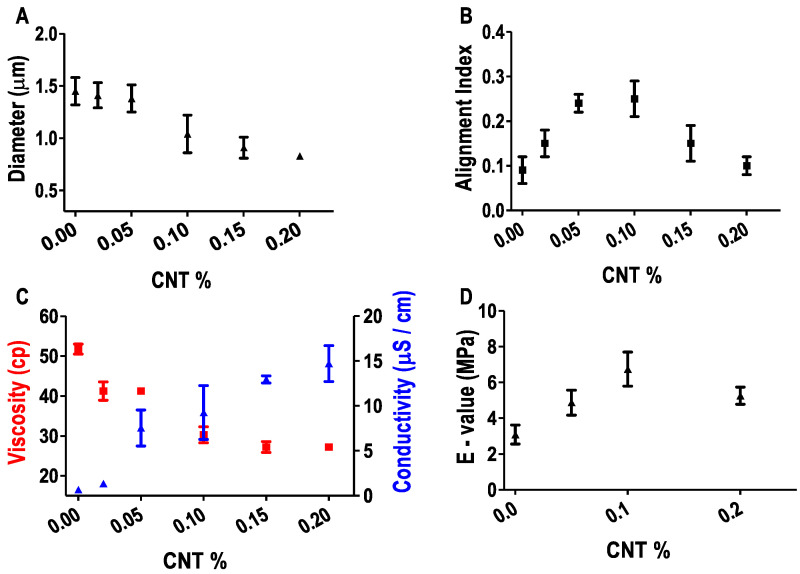
Variation of fiber properties with CNT percentage. (**A**) Fiber diameter derived from AFM images; (**B**) Fiber alignment index derived from optical images; (**C**) Fiber conductivity and solution viscosity; (**D**) Fibers’ Young’s modulus, E, measured by AFM-based nano-indentation method.

AFM was applied to characterize fiber diameter. Ethanol post-treatment was found to induce an 8–17% decrease of fiber diameter due to the dehydration effect. For post-treated fibers, fiber diameter decreased with the increase of CNT percentage (Figure 3A). The mean diameter of SF, SF-CNT 0.1%, and SF-CNT 0.2% fibers was 1.45 µm, 1.04 µm, and 0.83 µm, respectively.

Fiber alignment was characterized by 2D-FFT using bright-field images [2,6,21]. A higher alignment index indicates a better alignment. As shown in Figure 3B, addition of a minute amount of CNT improved fiber alignment significantly. The alignment index increased from 0.09 of SF fibers to 0.25 of SF-CNT 0.1% fibers. However, further addition of CNT caused a decrease of fiber alignment. The alignment of E-spun fibers is affected by both protein solution viscosity and conductivity. CNT is present as an insoluble impurity in SF solution. Given the same protein concentration (100 mg/mL), the addition of increased CNT led to a decrease of solution viscosity from 51.75 cP of SF to 27.2 cP of SF-CNT 0.2%, and is expected to decrease fiber alignment. On the other hand, the addition of CNT increased SF solution conductivity, which facilitated the ejection of the jet stream under a high voltage during electrospinning. Meanwhile, the fiber conductivity was found to increase from 0.675 µS/cm of SF to 9.26 µS/cm of SF-CNT 0.1% and 14.71 µS/cm of SF-CNT 0.2%. The resulting fibers demonstrated increased stiffness and conductivity, both positively impacting fiber alignment. The interplay of the opposing effects is accountable for the optimum fiber alignment in SF-CNT 0.1% fibers. Note that the reduced fiber diameter with CNT addition is attributed to the reduced viscosity of the electrospinning solution.

The effect of CNT on fibers’ stiffness was examined by AFM-based nanoindentation method. As shown in Figure 3D, with the increase of CNT percentage, the Young’s modulus (E-value) increased from 3.09 MPa for SF fibers to 6.74 MPa for SF-CNT 0.1% fibers. A higher percentage of CNT led to a decrease of fiber stiffness, likely due to the reduced fiber quality.

Taken together, SF-CNT 0.1% fibers outperform other fiber types concerning fiber diameter, mechanical property and alignment, mimicking aligned fibrous collagen in extracellular matrices of native tissues to support cell functions. They also demonstrated good conductivity, which will be exploited to mediate electrical stimulation of fibroblasts. Thus, SF-CNT 0.1% fibers are chosen in the rest cell studies.

### 3.4. Biocompatibility of SF-CNT Fibers

**Figure 4:**
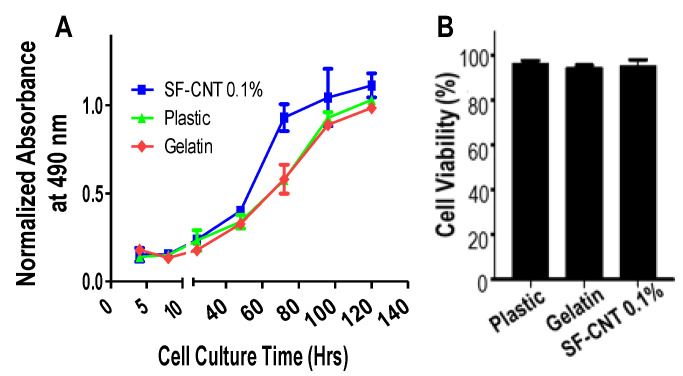
Biocompatibility of SF-CNT 0.1% fibers in supporting cell culture. (**A**) Cell proliferation profile. (**B**) Cell viability. Plastic and gelatin coated substrates were used as controls.

While CNTs can increase the conductivity of SF fibers, an excess amount of CNT is known to cause cytotoxicity [25]. The proliferation profile of cells cultured on SF-CNT 0.1% fibers was examined in comparison to that of cells cultured on plastic (Petri dish) and gelatin, the common cell culture substrates. As shown in Figure 4A, cells on SF-CNT fibers proliferated at a similar rate as cells on plastic and gelatin in the first 48 h, and at a slightly higher rate afterward. The proliferation curves plateaued on Day 4 for cells on all substrates, indicating cell confluency. On the other hand, cell viability remained high on SF-CNT 0.1% fibers (Figure 4B). It suggests that the addition of a minute amount of CNT caused negligible level of cytotoxicity.

In combination with the post-treated fibers’ aqueous stability and enzymatic degradability, SF-CNT 0.1% fibers are suitable for supporting long-term cell culture and being used as tissue engineering scaffolds in vivo.

### 3.5. Fibroblast Response to SF-CNT Mediated Electrical Stimulation

**Figure 5:**
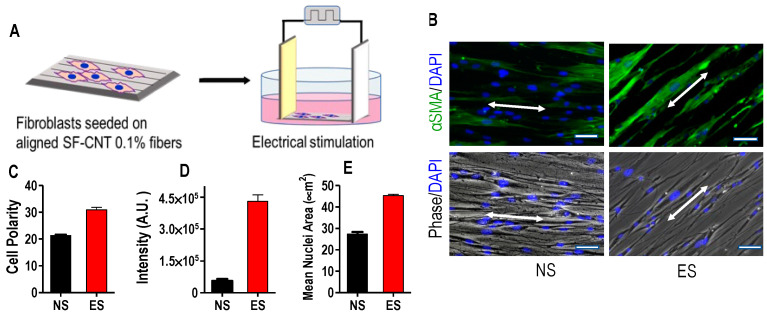
Cell response to electrical stimulation. (**A**) Scheme of electrical stimulation of fibroblasts on aligned SF-CNT fibers. (**B**) Phase and immunofluorescent images illustrating cell polarization and staining of α-SMA (green) and nuclei (blue) under non-stimulation (NS) and electrical stimulation (ES) conditions. Bar size: 250 µm. The arrows indicate the direction of fiber alignment. (**C**–**E**) Cell polarity (**C**), fluorescence intensity of α-SMA staining (**D**), and mean nuclear area (**E**) derived from images of cells grown on SF-CNT-0.1% fibers under NS and ES conditions. The difference of NS vs. ES is statistically significant: *p* < 0.0001 for cell polarity and mean nuclear area; *p* < 0.005 for α-SMA expression.

To investigate the modulation effect of the SF-CNT fibers, fibroblasts were seeded on fibers, cultured for 24 h, then subjected to electrical stimulation (ES) by applying an electric field in the direction of fiber alignment (Figure 5A). Immunostaining of α-smooth muscle actin (α-SMA), a marker protein of myofibroblasts, was carried out in the presence and absence of ES. As shown in Figure 5B, both stimulated and unstimulated cells were polarized in the direction of fiber alignment, and the stimulated cells expressed α-SMA at a significantly higher level. Quantitative analysis was carried out, and the results are shown in Figure 5C–E. Electrical stimulation boosted fibroblasts’ α-SMA expression by 9.49 folds, accompanied by a higher level of cell polarization and a noticeable increase of the size of nuclei. Additionally, the actin filaments in the elongated cytoskeleton were uniform, parallel to each other, and orderly aligned. These results consistently imply that the fibroblasts were activated by ES and transformed into myofibroblasts, which are highly contractile and are the phenotype to synthesize collagen actively.

**Figure 6:**
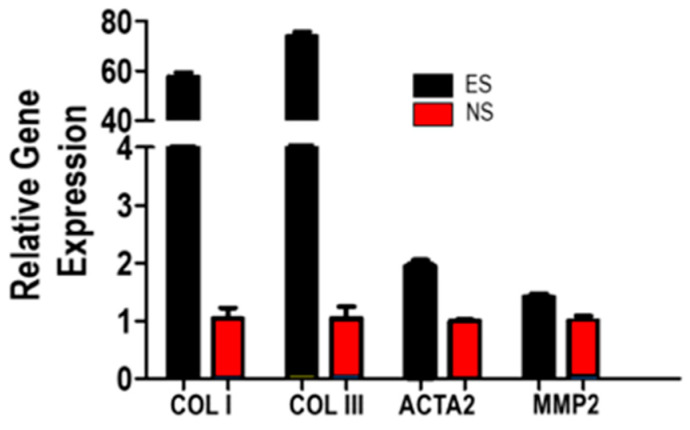
Gene expression profile of fibroblasts grown on SF-CNT fibers under ES (black) in reference to the expression level of each gene under NS condition (red). Error bars indicate standard error. The difference of ES vs. NS is statistically significant: *p* < 0.001 for COLI, COLIII and ACTA2; *p* < 0.015 for MMP2.

The effect of electrical stimulation on fibroblasts’ collagen productivity was examined by RT-qPCR analysis. As shown in Figure 6, the SF-CNT mediated electrical stimulation boosted the cells’ COLI and COLIII expression levels by 58 and 74 folds, respectively. Meanwhile, the expression levels of ACTA2 (for α-SMA) and MMP2 were increased by 2.0 and 1.4 folds, respectively. Both ACTA2 and MMP2 are associated with fibroblast activation [25]. In an extracellular matrix, fibrillar COLI offers high tensile strength, whereas COLIII is found alongside COLI in tissues that require flexibility and distension [18,30]. A matrix with a high COLI/COLIII ratio, as in the case of collagen in prolapse patients’ pelvic floor connective tissues, is correlated with stiffer collagen fibers [19]. Grown on the stiff, aligned SF-CNT fibers, the fibroblasts responded to ES by synthesizing more COLIII than COLI to remodel the matrix and attain a softer substrate with reduced COLI/COLIII ratio (Figure S3). The result implies that ES can not only augment collagen synthesis, but also modulate matrix biomechanics.

## 4. Discussion

In this work, we applied electrospinning technique to generate SF-CNT composite fibers by incorporating a minute amount of CNT in silk fibroin, an abundant and easy-to-process natural biopolymer. The as-spun fibers were found to quickly dissolve in water regardless of CNT composition. This is in contrast to native SF’s high aqueous stability, and is ascribed to the predominant α-helix and random coil secondary structures in the reconstituted protein fibers, evidenced by the signature peaks in FTIR and Raman spectra (Figure 2 and Figure S2). In native silkworm SF fibers, water soluble silk I regions and crystalline silk II regions co-present [8,31]. Silk II regions are dominant in SF fibers. They comprise GAGAGS, GAGAGY, GAGAGA or GAGYGA repeats, which form stable anti-parallel β-sheet crystallites with neighboring segments linked by strong hydrogen bonds. These crystalline domains are hydrophobic and responsible for the mechanical strength and aqueous stability of SF fibers [8,13,32]. Silk I regions consist of α-helices, random coils and amorphous regions with non-repetitive amino acid sequences. They are more hydrophilic and contribute to the elasticity and flexibility of SF fibers [13,33]. To enhance aqueous stability of the reconstituted E-spun fibers, we applied ethanol vapor treatment to induce the helix/coil to β-sheet secondary structural transition [34]. It is known that ethanol can act as a plasticizer to remove intermolecularly bound water molecules, facilitating hydrogen bond formation between peptides that favors their rearrangement into β-sheet crystallites [35]. The treatment was effective for both SF and SF-CNT fibers, and increased β-sheet conformation by 2.9 folds and 2.6 folds, respectively (Figure 2). Consequently, the fibers’ aqueous stability markedly improved, making them suitable to serve as tissue culture scaffolds. It was evident in our study that the fibers remained quite stable in supporting cell culture over three months.

While the conductivity of protein fibers was expected to increase with the added CNT, it is remarkable that a low dose CNT (0.1%) had increased the SF fiber conductivity by 13.6 folds. With its large surface area and hydrophobic nature, CNTs would preferentially interact with proteins’ hydrophobic domains that are present in the interior of a protein fiber. The protein-CNT fibers were stabilized by hydrogen bonds and electrostatic interaction due to the carboxyl groups on the oxidized CNTs and the positively charged amino acids in SF proteins. Putatively, the dominance of highly organized, hydrophobic β-sheet nanocrystals in post-treated fibers facilitated the SF-CNT interaction, effectively coaxed the CNT alignment along the fiber axis [36] and substantially increased the fiber conductivity. The addition of a higher amount of CNT did not significantly increase the conductivity and stiffness of SF fibers (Figure 3). Due to the distinctive mechanical properties of CNT and proteins, while a proper percentage of CNT can reinforce the strength and stiffness of the SF fibers, a higher percentage of CNT likely disrupts the protein fiber’s integrity attributing to the structural defects, misalignment and impaired fiber mechanics (Figure 1 and Figure 4). The decreased SF solution viscosity with CNT addition also contributed to the reduced fiber alignment due to the formation of small and unstable Taylor cones during electrospinning [37]. In native tissues, extracellular matrix proteins are locally aligned, providing physical cues to support cell functions. Concerning fiber structure, mechanics, conductivity, dimension, alignment and biocompatibility, SF-CNT 0.1% fibers outperformed other fiber types examined in this work.

It is known that the morphology and mechanical properties of a scaffold have significant impacts on cell behaviors [2,4,6]. We and others reported that, a stiff, aligned fibrous scaffold can polarize and activate fibroblasts to remodel the matrix [4,5,6,38]. An ES amplifies the effect [6,7,39,40]. Phenotypically, we observed ES invoked increase of cell polarization leading to a greater level of cytoskeletal tension and contractility to promote a more effective fibroblast to myofibroblast conversion (Figure 5 and Figure 6). This is evidenced by increased expression of α-SMA in ES cells. Myofibroblasts contract using α-SMA-myosin complexes [39]. The mechanically strong and electrically conductive SF-CNT fibers are hard to deform or break when the cells migrate and contract on them, therefore, can mediate the mechanotransductive and electrical stimulation signals more efficiently to activate TGFβ1 signaling for boosting fibroblasts’ collagen synthesis [40]. It is supported by the increased ACTA2 and MMP2 expressions in ES cells (Figure 6), which are known to activate TGFβ1 signaling [41,42].

Here, with aligned SF-CNT 0.1% fibers and 30 min ES, we achieved fibroblast activation and boosted COLI and COLIII production by 58 folds and 74 folds, respectively, when compared to that of NS cells grown on the same matrix (Figure 6). We chose to use primary vaginal fibroblasts of a stage III POP patient in this study. Fibroblasts of POP patients were characterized by reduced COLI and COLIII expression in comparison to that of non-POP individuals [6]. Additionally, the synthesized collagen is at an abnormally high COLI to COLIII ratio, leading to a loose and fragile collagen fiber network inadequate to support the pelvic organs [17,18,19]. The deficit fibroblasts are accountable for the connective tissue failure. Our approach has shown that patients’ fibroblasts can be remediated to boost collagen productivity dramatically. Additionally, the impaired COLI/COLIII ratio can be renewed. We achieved a similar level of augmentation of collagen synthesis in a previous study, in which a 6 h ES was required when the stimulation was mediated by CNT incorporated fibers of engineered spider silk protein [6]. It is prominent that remedy of patients’ fibroblasts can be achieved with a significantly reduced ES time and the use of inexpensive SF scaffolds.

Severe stages of POP often require reconstructive pelvic surgery by employing permanent or degradable meshes made from materials of synthetic or biological origin [43,44,45]. Concerns over high rates of complications, such as erosions, pain, infections, vaginal shrinkage, poor biocompatibility or lack of long-term effects undermined their usage. Additionally, the constructs provide physical support to relieve the stress of the local tissue and lift up the prolapsed organs, but do not rectify the aberrant biophysical and biochemical properties of collagen for tissue regeneration [19]. Availability of more management options and affordable treatments are highly desirable. Our approach offers restoration of the biological function of POP patients’ fibroblasts. The transformed cells can be resupplied to patients for reviving the tissue function. The use of autologous vaginal wall fibroblasts to treat vaginal wall connective tissue disorder is safe, simple and inexpensive, and is expected to be an economically favorable and clinically relevant alternative to reconstructive surgery or stem cell therapy.

## Data Availability

The data presented in this study are available on request from the corresponding author.

## Supplementary Materials

The following are available online at https://www.mdpi.com/article/10.3390/polym15010091/s1, Figure S1: Deconvolution and peak fitting of normalized FTIR spectra in amide I region; Figure S2: Raman spectra of as-spun and ethanol vapor treated SF-CNT 0.1% fibers; Figure S3: Effect of SF-CNT fibers and ES on COLI/COLIII ratio. Supplementary Material reference citations [3,23,24,26,46,47,48,49,50,51].
