# Supplementary material for: Electrospun Silk Fibroin-CNT Composite Fibers: Characterization and Application in Mediating Fibroblast Stimulation
Source: Polymers (Basel). 2022 Dec 26;15(1):91. doi: 10.3390/polym15010091 (PMC9824115; doi:10.3390/polym15010091)
Supplement: Supplementary file 1 [file polymers-15-00091-s001.zip › polymers-2101809-supplementary.pdf]

Supplementary Materials

# Electrospun Silk Fibroin-CNT Composite Fibers: Characterization and Application in Mediating Fibroblast Stimulation

**Rathnayake A. C. Rathnayake, Shinhae Yoon, Shuyao Zheng, Elwin D. Clutter, Rong R. Wang***

Department of Chemistry, Illinois Institute of Technology, Chicago, IL, USA

*Correspondence: wangr@iit.edu (R. R. Wang); Tel:1-312-567-3121

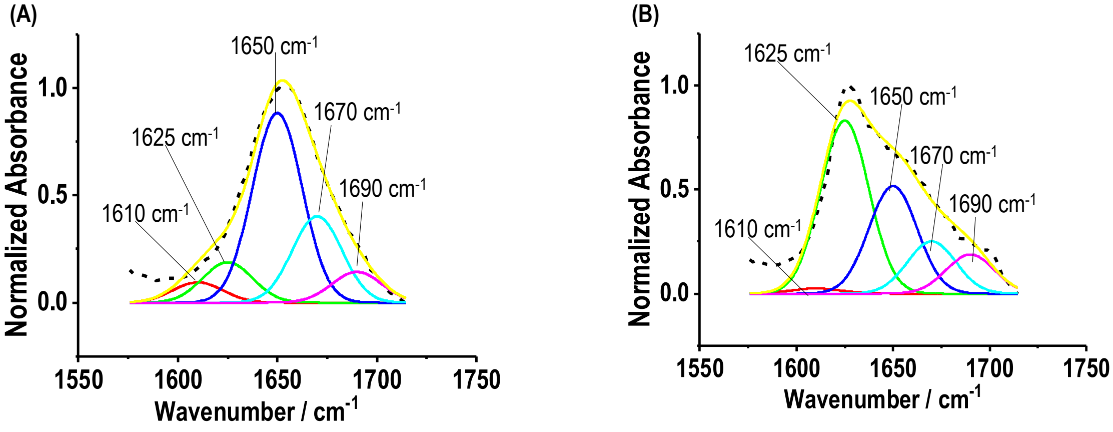


**Figure S1**. Deconvolution and peak fitting of normalized FTIR spectra in amide I region of as-spun SF fibers (A) and ethanol vapor treated SF fibers (B). The original spectra are black dashed. Each spectrum was fitted into 5 Gaussian peaks. Upon deconvolution, the best overlap of the reconstructed spectrum (yellow) with the original spectrum was obtained.

From the entire ATR-FTIR spectrum, amide I region (1585-1710 $cm^{-1}$) was chosen for quantitative analysis of secondary structures of SF and SF-CNT fibers before and after ethanol vapor treatment. Deconvolution was carried out using OriginLab software to fit Gaussian peaks using the following peak assignments: 1610 $cm^{-1}$ (turns), 1625 $cm^{-1}$ (β-sheet), 1650 $cm^{-1}$ ($\alpha$- helix / random coil), 1670 $cm^{-1}$ (β-turns), and 1690 $cm^{-1}$ (β-sheet) [1-2]. Peak FWHM were set to be identical (FWHM = 25 $cm^{-1}$) for all peaks in each spectrum, while allowing the center value and the intensity of each peak to vary for optimization of spectral deconvolution ($R^2 > 0.98$ for the best fitting). The percentage of secondary structural composition (in Figure 2E,F) was evaluated by integrating the area of each deconvoluted peak and then normalizing it to the total area of the amide I region of the fitted curve. Peaks at 1625 $cm^{-1}$ and 1690 $cm^{-1}$ were counted for β-sheets; peak at 1650 $cm^{-1}$ was counted for $\alpha$- helices and random coils.

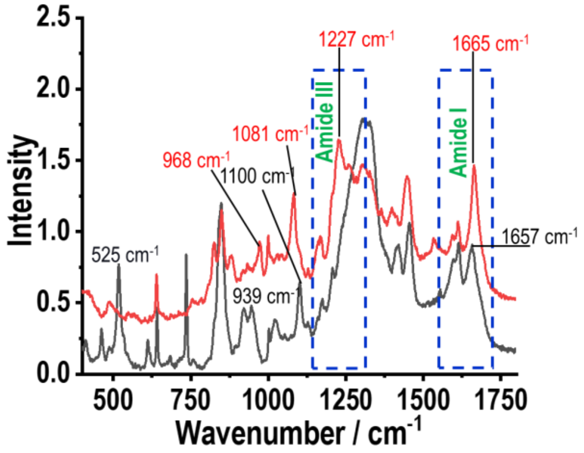


**Figure S2.** Raman spectra of as-spun (black) and ethanol vapor treated (red) SF-CNT 0.1% fibers.

The peaks associated with C-C stretches (900-1100 $cm^{-1}$), amide III (1200-1300 $cm^{-1}$), and amide I (1600-1700 $cm^{-1}$) are characteristic for protein secondary structures [3]. In as-spun fibers, the peak around 525 $cm^{-1}$ is characteristic for poly(alanine) in $\alpha$- helix conformation [4-5], and the peak at 1100 $cm^{-1}$ ($\upsilon$C-C) is associated to both $\alpha$-helix and random coil structures [6-8]. The 939 $cm^{-1}$ ($\upsilon$C-N) [9], 1270 $cm^{-1}$ (amide III)[9] and 1657 $cm^{-1}$ (amide I) [1, 4, 10] peaks are characteristic for $\alpha$- helix conformation. The presence of these peaks suggests that $\alpha$- helices and random coils are dominant secondary structures in the as-spun SF fibers. In contrast, ethanol treated SF fibers showed a strong peak at 1665 $cm^{-1}$ (amide I) as well as peaks at 1227 $cm^{-1}$ (amide III) and 965$cm^{-1}$, which identify β-sheet conformation [9]. Additionally, the bands at 525 $cm^{-1}$, 939 $cm^{-1}$ and 1100 $cm^{-1}$ disappeared. Collectively, the results confirmed that ethanol vapor treatment facilitates β-sheet formation.

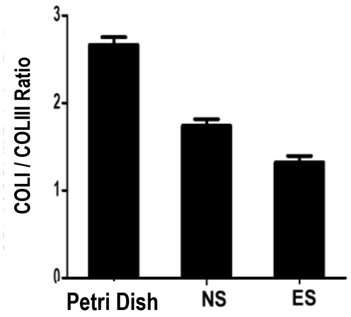


**Figure S3**. Effect of SF-CNT fibers and ES on COLI / COLIII ratio. The ratio is derived from COLI and COLIII gene expression in fibroblasts cultured on SF-CNT 0.1% fibers under NS and ES conditions. The ratio derived from cells cultured on Petri Dish, before trypsinized and reseeded on the fibers, was also derived for comparison.

The aligned, stiff SF-CNT fibers provided structural and mechanical cues to regulate the fibroblasts producing collagen at a reduced COLI/COLIII ratio, hence, remodel the surface to attain a softer matrix. In addition to boosting collagen synthesis, ES amplified the regulation effect by further lowering the COLI/COLIII ratio. The differencs are statistically significant: $p < 0.002$ for plastic vs. NS and plastic vs. ES; $p < 0.015$ for NS vs. ES.

# References:


1. Kreplak, L.; Doucet, J.; Dumas, P.; Briki, F., New aspects of the alpha-helix to beta-sheet transition in stretched hard alpha-keratin fibers. *Biophysical journal* **2004,** *87* (1), 640-7.
2. Zhu, B.; Li, W.; Chi, N.; Lewis, R. V.; Osamor, J.; Wang, R., Optimization of Glutaraldehyde Vapor Treatment for Electrospun Collagen/Silk Tissue Engineering Scaffolds. *ACS omega* **2017,** *2* (6), 2439-2450.
3. Stephens, J. S.; Fahnestock, S. R.; Farmer, R. S.; Kiick, K. L.; Chase, D. B.; Rabolt, J. F., Effects of Electrospinning and Solution Casting Protocols on the Secondary Structure of a Genetically Engineered Dragline Spider Silk Analogue Investigated via Fourier Transform Raman Spectroscopy. *Biomacromolecules* **2005,** *6* (3), 1405-1413.
4. Rousseau, M.-E.; Beaulieu, L.; Lefèvre, T.; Paradis, J.; Asakura, T.; Pézolet, M., Characterization by Raman Microspectroscopy of the Strain-Induced Conformational Transition in Fibroin Fibers from the Silkworm Samia cynthia ricini. *Biomacromolecules* **2006,** *7* (9), 2512-2521.
5. Frushour, B. G.; Koenig, J. L., Raman scattering of collagen, gelatin, and elastin. *Biopolymers* **1975,** *14* (2), 379-91.
6. Zheng, S.; Li, G.; Yao, W.; Yu, T., Raman Spectroscopic Investigation of the Denaturation Process of Silk Fibroin. *Applied Spectroscopy* **1989,** *43* (7), 1269-1272.
7. Rousseau, M.-E.; Lefèvre, T.; Beaulieu, L.; Asakura, T.; Pézolet, M., Study of Protein Conformation and Orientation in Silkworm and Spider Silk Fibers Using Raman Microspectroscopy. *Biomacromolecules* **2004,** *5* (6), 2247-2257.
8. Asakura, T.; Ashida, J.; Yamane, T.; Kameda, T.; Nakazawa, Y.; Ohgo, K.; Komatsu, K., A repeated β-turn structure in Poly(Ala-Gly) as a model for silk I of Bombyx mori silk fibroin studied with two-dimensional spin-diffusion NMR under off magic angle spinning and rotational echo double resonance11Edited by M. F. Summers. *Journal of Molecular Biology* **2001,** *306* (2), 291-305.
9. Monti, P.; Freddi, G.; Bertoluzza, A.; Kasai, N.; Tsukada, M., Raman spectroscopic studies of silk fibroin from Bombyx mori. *Journal of Raman Spectroscopy* **1998,** *29* (4), 297-304.
10. Stuart, B. H., Infrared Spectroscopy of Biological Applications. In *Encyclopedia of Analytical Chemistry*.
